# Recessive Inheritance of Congenital Hydrocephalus With Other Structural Brain Abnormalities Caused by Compound Heterozygous Mutations in *ATP1A3*

**DOI:** 10.3389/fncel.2019.00425

**Published:** 2019-09-26

**Authors:** August A. Allocco, Sheng Chih Jin, Phan Q. Duy, Charuta G. Furey, Xue Zeng, Weilai Dong, Carol Nelson-Williams, Jason K. Karimy, Tyrone DeSpenza, Le T. Hao, Benjamin Reeves, Shozeb Haider, Murat Gunel, Richard P. Lifton, Kristopher T. Kahle

**Affiliations:** ^1^Department of Neurosurgery, School of Medicine, Yale University, New Haven, CT, United States; ^2^Department of Genetics, School of Medicine, Yale University, New Haven, CT, United States; ^3^Laboratory of Human Genetics and Genomics, The Rockefeller University, New York, NY, United States; ^4^Department of Computational Chemistry, University College London School of Pharmacy, London, United Kingdom; ^5^Department of Cellular and Molecular Physiology, School of Medicine, Yale University, New Haven, CT, United States; ^6^NIH-Yale Centers for Mendelian Genomics, School of Medicine, Yale University, New Haven, CT, United States; ^7^Yale Stem Cell Center, School of Medicine, Yale University, New Haven, CT, United States

**Keywords:** congenital hydrocephalus, *ATP1A3*, Na^+^/K^+^ ATPase, whole exome sequencing, genetics

## Abstract

**Background:**

*ATP1A3* encodes the α3 subunit of the Na^+^/K^+^ ATPase, a fundamental ion-transporting enzyme. Primarily expressed in neurons, *ATP1A3* is mutated in several autosomal dominant neurological diseases. To our knowledge, damaging recessive genotypes in *ATP1A3* have never been associated with any human disease. *Atp1a3* deficiency in zebrafish results in hydrocephalus; however, no known association exists between *ATP1A3* and human congenital hydrocephalus (CH).

**Methods:**

We utilized whole-exome sequencing (WES), bioinformatics, and computational modeling to identify and characterize novel *ATP1A3* mutations in a patient with CH. We performed immunohistochemical studies using mouse embryonic brain tissues to characterize *Atp1a3* expression during brain development.

**Results:**

We identified two germline mutations in *ATP1A3* (p. Arg19Cys and p.Arg463Cys), each of which was inherited from one of the patient’s unaffected parents, in a single patient with severe obstructive CH due to aqueductal stenosis, along with open schizencephaly, type 1 Chiari malformation, and dysgenesis of the corpus callosum. Both mutations are predicted to be highly deleterious and impair protein stability. Immunohistochemical studies demonstrate robust *Atp1a3* expression in neural stem cells (NSCs), differentiated neurons, and choroid plexus of the mouse embryonic brain.

**Conclusion:**

These data provide the first evidence of a recessive human phenotype associated with mutations in *ATP1A3*, and implicate impaired Na^+^/K^+^ ATPase function in the pathogenesis of CH.

## Introduction

Congenital hydrocephalus (CH) is the most common reason for brain surgery in children and affects 1 in 1,000 newborns (Tully and Dobyns, 2014; Kahle et al., 2016). CH is characterized by ventriculomegaly, defined as dilation of cerebral ventricles, and thought to be secondary to impaired cerebrospinal fluid (CSF) homeostasis. Consequently, CH is treated by lifelong neurosurgical shunting with high complication rates and morbidity. The lack of satisfactory treatments highlights our incomplete understanding of CH pathogenesis (Kahle et al., 2016). There is a need to identify CH disease-causing genes, given that 40% of CH cases is estimated to have a genetic etiology (Haverkamp et al., 1999). Despite significant efforts to identify CH genes, including a recent whole-exome sequencing (WES) (Furey et al., 2018) study, the majority of CH cases remain idiopathic, underscoring the need for continued gene discovery.

*ATP1A3* encodes the α3 subunit of the Na^+^/K^+^ ATPase, a fundamental enzyme that regulates ion homeostasis by maintaining ionic gradients across the plasma membrane (Clausen et al., 2017). *ATP1A3* is highly expressed in neurons of the adult rodent brain (McGrail et al., 1991; Pietrini et al., 1992; Bottger et al., 2011) and mutations in the gene have been implicated in three autosomal dominant Mendelian diseases, including alternating hemiplegia of childhood (AHC) type 2 (Rosewich et al., 2012), CAPOS syndrome (Demos et al., 2014), and Dystonia-12 (Anselm et al., 2009). Knockdown of *Atp1a3* in zebrafish (Doganli et al., 2013) results in hydrocephalus; however, no known association exists between *ATP1A3* and human CH.

Here, we present the first case of obstructive CH with aqueductal stenosis and other structural brain abnormalities associated with recessive compound heterozygous mutations in *ATP1A3*.

## Materials and Methods

### Patient/Family Information

**FIGURE 1:**
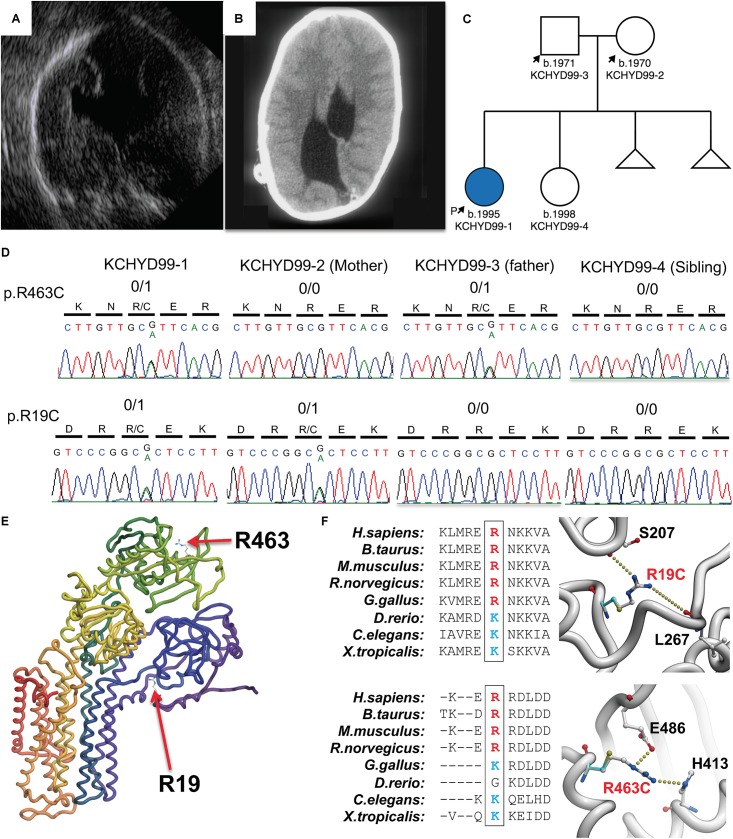
Damaging, protein-altering compound heterozygous *ATP1A3* mutations in human congenital hydrocephalus and other structural brain defects. Pre-natal ultrasound **(A)** and postnatal computed tomography **(B)** images demonstrating asymmetric ventriculomegaly, agenesis of the corpus callosum, schizencephaly, and aqueductal stenosis. **(C)** Patient pedigree with affected individual denoted in blue. **(D)** DNA chromatograms depicting the maternally inherited c.G55A (p.R19C) and paternally inherited c.G1387A (p.R463C) compound heterozygous mutations in *ATP1A3*, encoding the α3 catalytic subunit of the Na^+^/K^+^ ATPase. **(E)** Mutation locations in canonically folded wild-type α3 subunits. **(F)** Evolutionary conservation across multiple species showing conserved positively charged amino acid side chain and hydrogen bonding by the positively charged side chains of Arg19 and Arg463 lost due to mutation of these residues.

The patient is a 23-year-old Caucasian female of European descent. CH was diagnosed on prenatal ultrasound at 18 weeks gestation (Figure 1A). MR imaging captured immediately after delivery demonstrated marked asymmetric obstructive hydrocephalus secondary to aqueductal stenosis. The patient underwent ventriculoperitoneal shunt placement at birth with three subsequent surgical shunt revisions and was further diagnosed via MRI and computed tomography (Figure 1B) with craniosynostosis, open lip schizencephaly, type 1 Chiari malformation, dysgenesis of the corpus collosum, and learning disability. Routine genetic testing (FISH, microarray) was negative. The patient’s mother reports two previous miscarriages. The mother reports a medical history of hyperthyroidism while the father reports a history of hypertension and anxiety. There are no known medical problems that run in the family on either the maternal or paternal side. The patient has one phenotypically normal sister. Institutional review board approval was obtained from the Yale University Human Investigative Committee, and all participants provided written informed consent.

### Exome Sequencing and Analysis

To identify potential disease-causing variants in this patient, we performed WES on the affected individual and her parents. Targeted capture was performed using the xGEN Exome Research Panel v1.0 (IDT) followed by DNA sequencing on the Illumina HiSeq 4000 System. Sequence metrics are shown in Supplementary Table S1. Sequence reads were mapped to the reference genome (GRCh37) with BWA-MEM and further processed using the GATK Best Practices workflows (McKenna et al., 2010; Van der Auwera et al., 2013; 1000 Genomes Project Consortium, 2015) as previously described (Jin et al., 2017). Single nucleotide variants and small indels were called with GATK HaplotypeCaller and annotated using ANNOVAR (Wang et al., 2010), ExAC (v3), and gnomAD (v2.1.1) (Lek et al., 2016). The MetaSVM algorithm was used to predict deleteriousness of missense variants (Dong et al., 2015).

We filtered recessive variants for rare [minor allele frequency (MAF) ≤ 10^–3^ across ExAC and gnomAD] homozygous and compound heterozygous variants that exhibited high quality sequence reads. Only loss-of-function (LoF; including nonsense, frameshift, or canonical splice disruptions), D-Mis (MetaSVM-deleterious), and non-frameshift indels were considered potentially damaging to the disease. For dominant variants, we assessed for rare (MAF ≤ 2 × 10^–5^) and damaging variants (LoF or D-Mis). Finally, false positive variants were excluded by *in silico* visualization followed by Sanger sequencing validation.

### *In silico* Modeling

The sequence of the human α3 subunit of the Na^+^/K^+^ ATPase (*ATP1A3*) was taken from the Uniprot (P13637). The crystal structures of the Pig Sodium/potassium-transporting ATPase subunit α(PDB id 4RET and 3WGV) exhibit 86% sequence identity with human ATP1A3 (residues 1-1013). The structure of 4RET and 3WGV were used as templates to construct a homology model of the human ATP1A3 using MODELLER (Fiser and Sali, 2003). Conserved Arginine residues are present at the equivalent positions of R19 and R463 in both human and pigs. The spatial orientations of the side chains in the templates from pig were used to model the side chains of R19 and R463. A total of twenty models were built and subjected to restrained energy minimization to relieve any steric clashes between the side chains and the nucleic acid. The stereo-chemical parameters were analyzed using PROCHECK and PROSA (Wiederstein and Sippl, 2007) and the final model was chosen based on the on the basis of the lowest Cα RMSD value after superimposition on the template structure (0.9 Å). This lies within the permitted range for accurate homology model construction for sequence identity in the high range (>80%) (Fiser, 2010). The mutants, R19C and R463C, were constructed and the free energy of change calculated (ΔΔG) *in silico* using the ICM mutagenesis program (Laskowski et al., 1993)^1^.

### Immunohistochemistry

WT mouse embryos of C57/BL6 background were harvested at embryonic day 15.5. Brains were dissected and fixed in 4% PFA in PBS overnight at 4°C then cryoprotected in 30% sucrose in PBS for 48 h. Brains were then mounted in frozen OCT blocks and sectioned by cryostat at 25 μm in the coronal plane. Sections were mounted on microscope slides and stored in −80°C until use. To begin staining, slides containing brain sections were first thawed at room temperature, then washed in 0.5% PBST. Sections underwent antigen retrieval using citrate buffer, then washed in 0.5% PBST. Sections were blocked in 10% normal goat serum (NGS) in PBST at room temperature for 1 h, then incubated with primary antibodies diluted in 2.5% NGS in PBST at 4°C overnight. The primary antibodies were rabbit polyclonal anti-Atp1a3 (1:500 dilution) (Pietrini et al., 1992), mouse monoclonal anti-NeuN (1:250, MAB377, Millipore), and mouse monoclonal anti-Sox2 directly conjugated with Dylight 550 (1:250, MA1-014-D550, Invitrogen). Following primary antibody incubation, sections were washed and incubated with Alexa Fluor-conjugated secondary antibodies (1:500 in 2.5% NGS in PBST) for 1 h at room temperature. Following the final wash, slides were coverslipped with Prolong Gold Antifade mounting medium. For negative control, the primary antibody solution (containing anti-Atp1a3) was blocked with the immunizing peptide sequence (gdkkddsspkksc) (Pietrini et al., 1992). Images were acquired using the Zeiss LSM 880 confocal microscope or the Aperio digital scanner microscope. All experiments were done in accordance with the regulations set forth by the Yale University animal care and use committee.

## Results

Compound heterozygous mutations in *ATP1A3* were identified in the affected individual. A maternally inherited single nucleotide G to A variation was identified at cDNA NM_152296 position 55 in exon 2 (gnomAD MAF = 6.4 × 10^–5^), corresponding to the amino acid substitution p. Arg19Cys in the α3 subunit. A paternally inherited single nucleotide G to A variation was identified at cDNA position 1387 in exon 11 (gnomAD MAF = 4.5 × 10^–4^), corresponding to the amino acid substitution p.Arg463Cys in the α3 subunit (Figures 1C,D). *ATP1A3* is very intolerant to both loss-of-function mutations (pLI = 1) and missense variants (mis_z = 6.33) per gnomAD. No homozygous loss-of-function mutations or homozygous damaging missense mutations in *ATP1A3* have been reported in gnomAD. Both variants are predicted deleterious per MetaSVM. CADD scores for the p.Arg19Cys and p.Arg463Cys variants were 34 and 24.8, respectively. Conservation of positively charged amino acid residues (arginine and lysine) was observed across species (Figure 1F).

*In silico* modeling of p.Arg19Cys and p.Arg463Cys mutations shows disruptive effects on protein stability. The side chain of arg-19 forms interactions with the backbone atoms of Ser-207 and Leu-267. This interaction is lost when Arg-19 is mutated to a cysteine with a corresponding energy penalty (ΔΔ*G* = 2.3 kcal/mole, Figures 1E,F). Similarly, the side chain of Arg-463 makes strong hydrogen bond interactions with the side chain of Glu-486 and His-413. These strong interactions are lost when Arg-463 is mutated to a Cys (ΔΔ*G* = 3.2 kcal/mole, Figures 1E,F).

**FIGURE 2:**
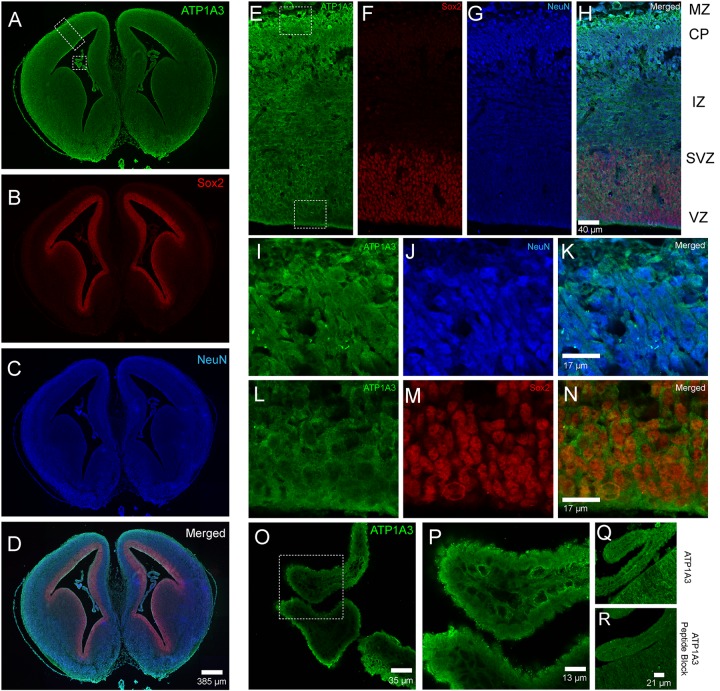
Expression of Atp1a3 in the embryonic mouse brain at E15.5. Fluorescent images taken at low magnification showing expression of **(A)** Atp1a3, **(B)** Sox2, **(C)** NeuN, and **(D)** all channels merged in the embryonic mouse brain. Dotted boxes in panel A identify the cortex and the choroid plexus. High magnification cortical expression of **(E)** Atp1a3, **(F)** Sox2, **(G)** NeuN, and **(H)** all channels merged. MZ – marginal zone, CP – cortical plate, IZ – intermediate zone, SVZ – subventricular zone, VZ – ventricular zone. Dotted boxes in panel **(E)** shows the cortical plate and ventricular zone. **(I–K)** High magnification expression of Atp1a3 in NeuN^+^ cells in the CP. **(L–N)** Expression of Atp1a3 in Sox2^+^ cells in the ventricular zone. **(O,P)** High-magnification expression of Atp1a3 in the choroid plexus. **(Q)** Brain sections incubated with Atp1a3 primary antibody alone or **(R)** Atp1a3 primary antibody blocked with immunizing peptide.

The association of *ATP1A3* mutations with CH and multiple other significant structural brain abnormalities implicate ATP1A3 in human brain development. Thus, we performed immunofluorescence studies in the embryonic mouse brain to characterize Atp1a3 expression. We stained histological brain sections collected from embryonic day 15.5 (E15.5) with an antibody against Atp1a3 (Pietrini et al., 1992) together with Sox2 [a marker of neural stem cells (NSCs)] (Ellis et al., 2004) and NeuN (a marker of differentiated neurons) (Mullen et al., 1992). Overall, Atp1a3 exhibits diffused and cytoplasmic expression throughout all cortical layers of the embryonic mouse brain (Figure 2). Co-localization studies showed that Atp1a3 is expressed in differentiated neurons at the cortical plate (CP) and in the NSCs at the ventricular zone (VZ) lining the lateral ventricles (Figures 2E–N). We also detected Atp1a3 expression in choroid plexus epithelial cells (Figures 2O,P). Furthermore, brain sections depicting the VZ and choroid plexus incubated with the Atp1a3 antibody blocked with the immunizing peptide showed minimal immuno-reactivity (Figures 2Q,R), demonstrating antibody specificity.

## Discussion

Atp1a3 expression is exclusive to neurons and highly expressed in inhibitory interneurons (Richards et al., 2007), where it plays a crucial role in electrophysiological functions including ion gradient maintenance (Arystarkhova et al., 2019), after hyperpolarization (Picton et al., 2017), and suppression of burst firing (Vaillend et al., 2002). Mice harboring loss of function mutations in *ATP1A3* exhibit increased seizure activity and neuronal hyperexcitability (Clapcote et al., 2009; Hunanyan et al., 2015) suggesting that *ATP1A3* is crucial to maintaining normal neurological function. Human mutations in *ATP1A3* have previously been discovered in a wide range of autosomal dominant neurological disorders (Heinzen et al., 2014), including encephalopathy with cerebellar ataxia (Sabouraud et al., 2019), AHC (Galaz-Montoya et al., 2019), rapid-onset dystonia Parkinsonism (ADP) (Anselm et al., 2009), cerebellar ataxia (Sabouraud et al., 2019), early-onset epilepsy (Ishihara et al., 2019), and autism spectrum disorder (Torres et al., 2018). Importantly, the majority of the mutations identified in AHC and RDP cluster in exons 8, 14, 17, and 18 (Rosewich et al., 2014), which are distinct from our variants (exon 2 and 11). To our knowledge, damaging recessive genotypes in *ATP1A3* have never been associated with any human disease.

We identified two germline mutations in *ATP1A3* (p. Arg19Cys and p.Arg463Cys), each of which was inherited from one of the patient’s unaffected parents, in a single patient with severe obstructive CH due to aqueductal stenosis, along with open schizencephaly, type 1 Chiari malformation, and dysgenesis of the corpus callosum. Both mutations are predicted to be highly deleterious and impair protein stability. Consistent with our results, morpholino knockdown of *Atp1a3* causes ventriculomegaly in zebrafish (Doganli et al., 2013), recapitulating the hydrocephalus phenotype in our patient. Together, these data provide evidence for a recessive inheritance of CH with aqueductal stenosis caused by *ATP1A3* compound heterozygous mutations.

Extending from previous work demonstrating expression of Atp1a3 in mature neurons (McGrail et al., 1991; Pietrini et al., 1992; Bottger et al., 2011), we found Atp1a3 to also be expressed in NSCs, differentiated neurons, and choroid plexus epithelial cells of the mouse embryonic brain. Our findings suggest two separate but not necessarily mutually exclusive mechanisms whereby *ATP1A3* compound heterozygous mutations may cause ventriculomegaly. First, hydrocephalus is classically thought to be a disorder of failed CSF homeostasis secondary to obstructed flow, increased secretion, or decreased absorption by the arachnoid villi (Kahle et al., 2016). The ion pump Na^+^/K^+^-ATPase is known to regulate CSF secretion in the choroid by maintaining an osmotic gradient of Na^+^ that drives the movement of water into the cerebral ventricles (Masuzawa et al., 1984; Fisone et al., 1995; Speake et al., 2001). The Na^+^/K^+^-ATPase is composed of three subunits: α, β, and γ (Holm et al., 2016). The catalytic activity of Na^+^/K^+^-ATPase has been attributed to the α subunit, which binds ATP, Na^+^, and K^+^. Thus, α subunit dysfunction due to p. Arg19Cys and p.Arg463Cys mutations described in this study can impair CSF homeostasis and thus drive the development of hydrocephalus.

Second, our finding of Atp1a3 expression in embryonic NSCs suggests a novel role of *ATP1A3* in regulating neural development. Thus, mutations that disrupt ATPA13 function may impair NSC regulation and suggest dysregulation of neural development, rather than failed CSF homeostasis, to be the primary pathogenic driver of human CH. Indeed, multiple lines of evidence from animals (Huang et al., 2007; Lessard et al., 2007; Carter et al., 2012; Roy et al., 2019) and humans (Guerra et al., 2015; Rodriguez and Guerra, 2017; Furey et al., 2018) suggest abnormal NSC development to be a primary driver of CH pathogenesis. This potential impact on the general process of neural development may not only underpin ventriculomegaly but also other developmental anomalies (type 1 Chiari malformation, corpus callosum dysgenesis, and intellectual disability) observed in our patient. In addition, the notable lack of dystonic, epileptic, or motor deficits in this patient suggests that the spectrum of *ATP1A3* linked diseases may be broader than previously acknowledged.

In sum, our findings provide the first association of human CH with recessive mutations in *ATP1A3*, setting the stage for future studies to better understand the role of *ATP1A3* in brain development and the pathogenesis of human CH.

## Data Availability Statement

The datasets generated for this study can be found in the dbGAP: phs000744.

## Ethics Statement

The studies involving human participants were reviewed and approved by Yale University Human Investigative Committee. The patients/participants provided their written informed consent to participate in this study.

## Author Contributions

AA designed the study, collected and analyzed the data, and wrote the manuscript. SJ and PD collected and analyzed the data, and wrote the manuscript. CF, CW, JK, TD, LH, and BR collected the data. XZ and WD collected and analyzed the data. SH analyzed the data. MG designed the study. RL designed the study and analyzed the data. KK designed the study, analyzed the data, and wrote the manuscript.

## Conflict of Interest

The authors declare that the research was conducted in the absence of any commercial or financial relationships that could be construed as a potential conflict of interest.

## Footnotes

1 www.molsoft.com
