# Supplementary material for: Recessive Inheritance of Congenital Hydrocephalus With Other Structural Brain Abnormalities Caused by Compound Heterozygous Mutations in ATP1A3
Source: Front Cell Neurosci. 2019 Sep 26;13:425. doi: 10.3389/fncel.2019.00425 (PMC6775207; doi:10.3389/fncel.2019.00425)
Supplement: Supplementary file 1 [file Table_1.DOCX]

| **Supplementary Table 1. Summary sequencing statistics for the KCHYD99 family** | |
| --- | --- |
| Category | Cases |
|  | (IDT; N=3) |
| Read length (bp) | 101 |
| # of reads per sample (M) | 60.7 |
| Median coverage at each targeted base (X) | 63.3 |
| Mean coverage at each targeted base (X) | 69.4 |
| % of all reads that map to target | 57.86% |
| % of all bases that map to target | 43.87% |
| % of targeted bases read at least 8x | 98.77% |
| % of targeted bases read at least 10x | 98.63% |
| % of targeted bases read at least 15x | 97.93% |
| % Mean error rate | 0.31% |
